# Comparison of gut microflora of donkeys in high and low altitude areas

**DOI:** 10.3389/fmicb.2022.964799

**Published:** 2022-09-26

**Authors:** Rong Guo, Shuer Zhang, Jianxing Chen, Wei Shen, Guoliang Zhang, Junjie Wang, Fali Zhang, Qingjie Pan, Taifeng Xie, Deqiang Ai, Jianbao Dong, Jiajia Suo, Yujiang Sun, Shuqin Liu

**Affiliations:** ^1^College of Animal Science and Technology, Qingdao Agricultural University, Qingdao, Shandong, China; ^2^Shandong Animal Husbandry General Station, Jinan, Shandong, China; ^3^College of Chemistry and Life Science, Chifeng University, Chifeng, Inner Mongolia, China; ^4^Gene Bank of Equine Genetic Resources, Qingdao, Shandong, China; ^5^College of Life Sciences, Qingdao Agricultural University, Qingdao, Shandong, China; ^6^Qinghai Sheep Breeding and Extension Service Center, Gangcha County, Haibei Prefecture, Qinghai, China; ^7^Department of Veterinary Medical Science, Shandong Vocational Animal Science and Veterinary College, Weifang, Shandong, China; ^8^Vocational College of Dongying, Dongying, Shandong, China

**Keywords:** donkey, gut microbes, altitude, 16S rRNA, metagenomic

## Abstract

Donkeys’ gut microbe is critical for their health and adaptation to the environment. Little research has been conducted on the donkey gut microbiome compared with other domestic animals. The Tibetan Plateau is an extreme environment. In this study, 6 Qinghai donkeys (QH) from the Tibetan Plateau and 6 Dezhou donkeys (DZ) were investigated, and the contents of 4 parts—stomach, small intestine, cecum, and rectum—were collected. 16S rRNA sequencing and metagenomic sequencing were used to analyze the composition and diversity of gut microbial communities in donkeys. The results showed that the flora diversity and richness of the hindgut were significantly higher than those of the foregut (*p* < 0.01), with no sex differences, and the community structure and composition of the same or adjacent regions (stomach, small intestine, cecum, and rectum) were similar. Besides, the flora diversity and richness of QH on the Tibetan Plateau were significantly higher than those of DZ (*p* < 0.05). The major pathways associated with QH were signal transduction mechanisms and carbohydrate transport and metabolism, and *Bacteroidales* were the major contributors to these functions. Our study provides novel insights into the contribution of microbiomes to the adaptive evolution of donkeys.

## Introduction

Donkeys have unique digestive characteristics; the biggest difference from ruminants is that donkeys are hindgut fermentation. The donkey has a well-developed hindgut structure with a length of more than 4.5 m and a volume of more than 110 l, which is approximately 16 times the volume of its foregut (Liu et al., 2019).

The animal gut tract has a complex microbial ecosystem, and the microorganisms in the gut were closely related to the life activities of the host, which may affect growth and metabolism, nutritional digestion, immunity, and the ability to resist invading pathogens (Egert et al., 2006; Edgar, 2013). Studies have shown that the region (He et al., 2018) and environment (Rothschild et al., 2018) were the main factors affecting gut microbes. The environment is one of the factors that cause and maintain the differences in and diversity of gut microbes in the hosts of different regions. The proportion of shared flora decreases exponentially with an increase in the distance from the host distribution area, resulting in a gradual increase in the difference in flora (Moeller et al., 2017). Monaghan et al. (2020) studied the structure, function, antibiotic resistance, and resistance to *Clostridioides difficile* infectious diarrhea of gut microbes in 105 urban and rural populations in central India and found that the composition of bacteria and viruses in the gut has obvious urban–rural differences, with geography having the greatest impact. Studies had shown that geographical conditions played a key role in shaping the diversity of the gut flora of iguanas, bats, and European fireflies (Lankau et al., 2012; Phillips et al., 2012; Sudakaran et al., 2012). Additionally, the research on factors influencing the gut flora of mice and fish had also found that hosts’ geographic conditions and diet affected the composition and structure of the gut flora (Sullam et al., 2012). Lorenc et al. (2014) used 454 pyrosequencing technology to sequence the 16S rRNA of 121 house mice in eight regions of Western Europe and found that geographical conditions were the main factors that determined the colonization pattern of the gut microbial community. These results indicate that microbiome interventions to improve clinical treatment should focus on geographical specificity.

The diversification of ecological types in China has created abundant donkey resources. More than 30 donkey species have been observed, such as the Tibetan Plateau Qinghai donkey (QH, in a plateau continental climate) and the Shandong Dezhou donkey (DZ, in a temperate monsoon climate in the North China Plain). Different regions often have different environmental conditions, and how host and symbiotic bacteria adapt to different environments has been investigated (Zhou et al., 2016). At this stage, although attention to the research on gut microbes has been increasing, few studies have investigated donkey gut microbes. In this study, a high-throughput 16S rRNA sequencing method combined with metagenomics was used to study the composition and structure of donkey gut microbes, as well as the commonality and characteristics of donkey gut microbes in different regions (Qinghai, Dezhou), laying a foundation for further research.

## Materials and methods

### Sample collection and DNA extraction in 16S rRNA analysis

A total of 12 healthy adult donkeys aged 5–10 years old with medium condition were used in this study: 6 Qinghai donkeys (QH, three males and three females) from the Qinghai region (altitude >3,000 m) and 6 Dezhou donkeys (DZ 3 males and 3 females) from Shandong Province (altitude <30 m). Both QH and DZ were stall-feeding with similar feeding management. After the donkeys were slaughtered, correctly separated the stomach, small intestine, cecum and rectum, fasten the joints of each segment with a rope, took samples from the middle of each segment, and took equal volumes of contents from the duodenum, jejunum and ileum, the contents were mixed as the small intestine contents. Fresh fecal contents from the stomach, small intestine, cecum, and rectum were collected and immediately stored in liquid nitrogen.

### 16S rRNA sequencing and analysis

Genomic DNA was extracted using a DNA extraction kit (Omega Bio-tek, Norcross, GA, United States) and measured on a 1% agarose gel by using a UV–Vis spectrophotometer (Thermo Fisher Scientific, Wilmington, United States). The hypervariable region V3–V4 of the bacterial 16S rRNA gene was amplified using fusion primers (F:5′-ACTCCTACGGGAGGCAGCAG-3′ and R:5′-GGACTACHVGGGTWTCTA AT-3′). PCR was performed in a total volume of 20 μl containing 4 μl 5 × TransStart FastPfu buffer, 2 μl 2.5 mmol/L dNTPs, 0.8 μl forward primer (5 μmol/L), 0.8 μl reverse primer (5 μmol/L), 0.4 μl TransStart FastPfu DNA Polymerase, 10 ng template DNA, and ddH_2_O up to 20 μl. PCR amplification was conducted as follows: initial denaturation at 95°C for 3 min; followed by 27 cycles of 95°C for 30 s, 55°C for 30 s, and 72°C for 45 s; and a final extension at 72°C for 10 min. The PCR products were extracted from a 2% agarose gel, and the purified amplicons were pooled in equimolar amounts and sequenced in a paired-end manner on the Illumina NovaSeq PE250 platform (Illumina, San Diego, United States). With the aim of obtaining high-quality clean reads, raw reads were demultiplexed and filtered by fastp version 0.20.0 (Chen et al., 2018) and merged by FLASH version 1.2.7 (Magoc and Salzberg, 2011) using the following criteria: reads with a length less than 50 bp, average base quality value less than 20, and N bases after shearing were removed. Next, filtered reads were assembled into raw tags according to the overlapping sequences of more than 10 bp, with a 0.2 mismatch. OTUs with a 97% similarity cut-off (Stackebrandt and Goebel, 1994; Edgar, 2013) were clustered using UPARSE version 7.1, and chimeric sequences were removed. The taxonomy of each OTU representative sequence was analyzed using RDP Classifier version 2.2 (Wang et al., 2007) against the 16S rRNA database (Silva v138), with a confidence threshold of 0.7.

### Metagenomic sequencing and analysis

On the basis of 16S rRNA sequencing results, 5 QH and 5 DZ rectal content samples were selected for metagenomic sequencing, and the extracted genomic DNA was submitted to Majorbio Bio-Pharm Technology Co., Ltd. (Shanghai, China). DNA fragments with an average size of approximately 400 bp were obtained using sonication. Paired-end sequencing was performed on an Illumina NovaSeq platform.^1^

Raw reads from metagenome sequencing were used to generate clean reads by removing adaptor sequences, trimming, and removing low-quality reads using fastp (Chen et al., 2018) (version 0.20.0). These high-quality reads were then assembled into contigs using MEGAHIT (Li et al., 2015) (https://github.com/voutcn/megahit, version 1.1.2), which used succinct de Bruijn graphs. Contigs with a length greater than or equal to 300 bp were selected as the final assembling result. Open reading frames (ORFs) in contigs were identified using MetaGene (Noguchi et al., 2006). Predicted ORFs with lengths greater than or equal to 100 bp were retrieved and translated into amino acid sequences by using the NCBI translation table. A non-redundant gene catalog was constructed using CD-HIT (Fu et al., 2012) (http://www.bioinformatics.org/cd-hit/, version 4.6.1) with 90% sequence identity and 90% coverage. After quality control, reads were mapped to the non-redundant gene catalog with 95% identity by using SOAPaligner (Li et al., 2008), and gene abundance in each sample was evaluated. Functional information was obtained: gene sequences were compared with those of the non-supervised orthologous groups (eggNOG) and KEGG databases. LEfSe analysis was performed to distinguish the functional components that had significant differential effects between the QH and DZ groups.

## Results

### 16S rRNA sequencing results for gut parts

#### Alpha diversity and species composition analysis

The 16S rRNA sequencing generated 2,348,518 reads in the original sequence, and the number of bases was 1,413,807,836 bp. After filtering, 2,348,518 reads and 984,132,676 bp were obtained. The average length of the effective sequences of all samples was 419 bp. There were 4,173 operational taxonomic units (OTUs), including 33 OTUs at the phylum level and 815 OTUs at the genus level.

**Figure 1:**
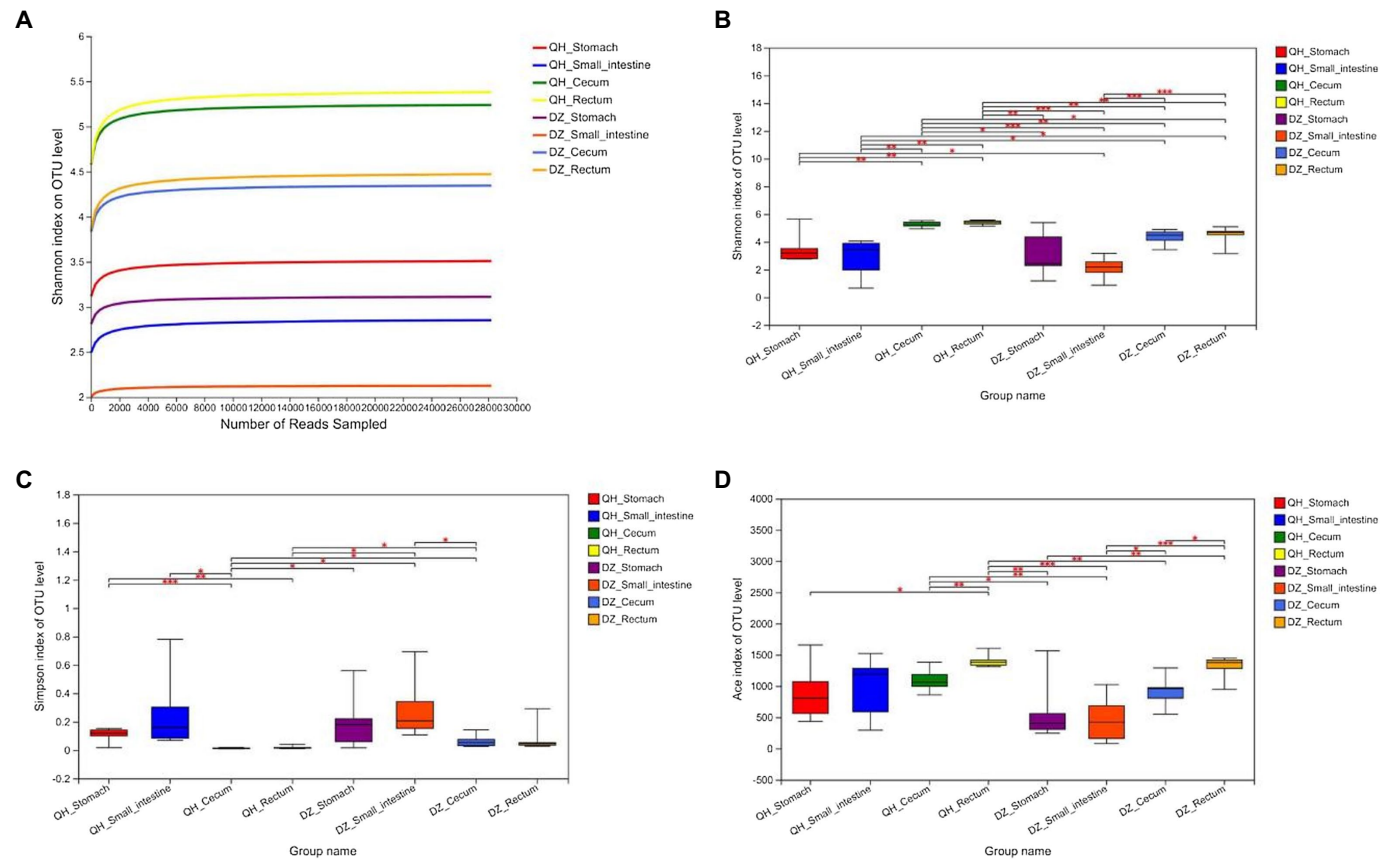
Alpha diversity analysis. **(A)** Sparse curve. Shannon’s exponential curve. The abscissa is the number of sequences; the ordinate is the OTU index. **(B–D)** Between-group *t*-test. Type I intervals represent the upper and lower limits of the index. The index difference test chart showed the significant difference between the selected two groups with significant differences (*p < 0.05, **p < 0.001, ***p < 0.01), the abscissa was the group name, and the ordinate was the index average of each group.

There was no evident difference between sexes in the same geographic location in the QH and DZ. The richness and diversity of the eight groups were shown in Figure 1. The Shannon index curve gradually flattened after rising, indicating that the sequencing data had reached saturation and could cover most species of the gut microbiome community (Figure 1A). The Shannon, Simpsoneven, and Ace indices showed significant differences among the stomach, small intestine, cecum, and rectum. The alpha diversity of QH was significantly higher than that of DZ, suggesting that the bacterial diversity of QH and DZ was significantly different (Figures 1B–D).

**Figure 2:**
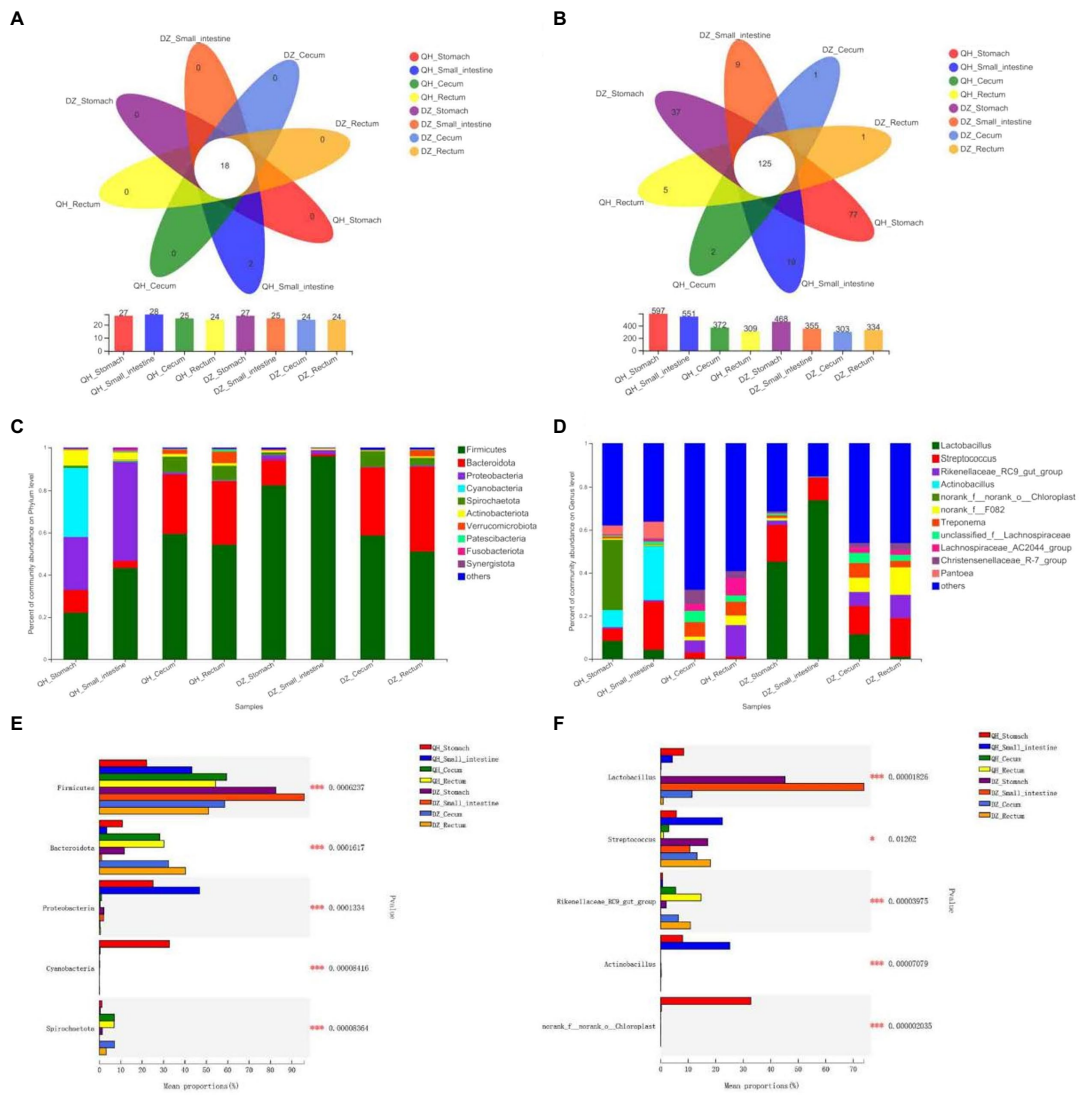
Composition of gut microbial community. Structure composition of related microbial communities in gut locations of phylum level **(A,C,E)** and genus level **(B,D,F)**. **(A,B)** Venn diagram. Numbers in the overlapping part represent the number of species shared by multiple groups, and the numbers in the non-overlapping part represent the number of species unique to the corresponding grouping. **(C,D)** Community column chart. The abscissa was the sample name, the ordinate was the proportion of species in the sample, the columns of different colors represented different species, and the length of the column represented the proportion of the species. **(E,F)** Multi-group comparison. The *X*-axis represented different groups, the boxes with different colors represented different groups, and the *Y*-axis represented the average relative abundance of a species in different groups.

At the phylum level, *Firmicutes* was the predominant phylum in all gut parts (the average proportion was 58.38%), and *Bacteroidetes* accounted for a large proportion in the cecum and rectum (36–58%) (Figures 2C,E). The proportion of *Firmicutes* was highest in the DZ-Small intestine and higher than that in the QH-Small intestine (*p* = 0.0370), QH-Rectum (*p* = 0.0400), and QH-Stomach (*p* = 0.0019). The proportion of *Bacteroides* in the cecum and rectum was higher than that in the stomach and small intestine in the QH and DZ (*p* = 0.0010) (Figure 2E). The proportion of *Proteobacteria* in QH-Stomach (25.13%) and QH-Small intestine (46.88%) was higher than that in other groups; *Cyanobacteria* accounted for the largest proportion of 32.77% in QH-Stomach, which was a potential biomarker to distinguish QH and DZ donkeys (Figures 2A,C,E). The proportion of *Spirobacteria* in the cecum and rectum was higher than that in the stomach and small intestine in QH and DZ (*p* = 0.0010), the proportion of *Spirobacteria* in QH-Small intestine was higher than DZ-Small intestine (*p* = 0.0480), and that in QH-Cecum was higher than DZ-Rectum (*p* = 0.0400) (Figure 2E). Notably, the QH-Small intestine had two unique phyla: *Caldisericota* and *Hydrogenedentes* (Figure 2A).

At the genus level, 276 bacteria were identified, of which were 125 species (45.29%) were present in all groups. Based on the identified genera, the species of the bacterial community was rich in the cecum and rectum of QH and DZ (Figures 2B,D). After comparative analysis, the *Lactobacillus* content in the DZ-Small intestine was the highest, the average relative abundance in the QH-Stomach and QH-Small intestine was lower than that in the DZ-Stomach and DZ-Small intestine (*p* = 0.0200), and QH-Cecum and QH-Rectum were less abundant than in the DZ-Cecum and DZ-Rectum (*p* = 0.0100). *Streptococcus* was most abundant in the QH-Small intestine and was significantly lower in the QH-Rectum than in the QH-Stomach (*p* = 0.0400) and DZ-Cecum (*p* = 0.0300). The number of *Rikenellaceae* was higher in the cecum and rectum than in the stomach and small intestine in QH and DZ and higher in QH-Rectum than in QH-Cecum (*p* = 0.0400). *Actinobacteria* were present in the QH-Stomach and QH-Small intestine; *chloroplasts* were present only in the QH-Stomach (*p* = 0.0010) and could be potential biomarkers that differentiate between QH and DZ donkeys (Figures 2D,F).

### Beta diversity analysis

**Figure 3:**
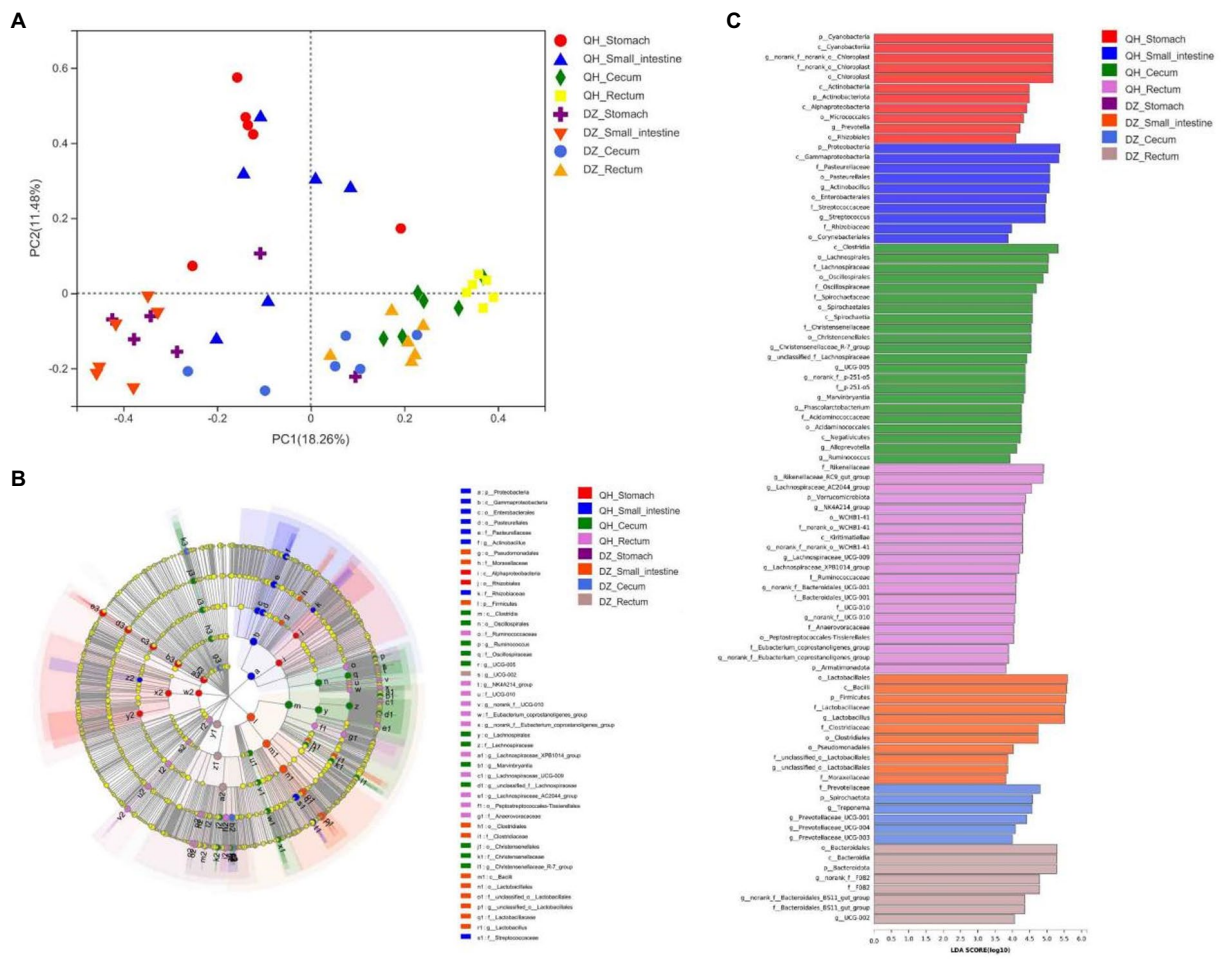
Beta diversity analysis. **(A)** PCoA picture. The *X*-axis and *Y*-axis represented the two selected primary axes, and the percentages represented the explanatory value of the differences in sample composition by the primary axes; points with different colors or shapes represented samples in different groups: the closer the two sample points, the more similar the species composition of the two samples. **(B)** LEfSe diagram. Nodes with different colors indicate microbial groups that were significantly enriched in the corresponding groups and have a significant impact on the differences between groups; light yellow nodes indicate microorganisms that have no significant differences in different groups or have no significant impact on differences between groups. **(C)** LDA diagram. The abscissa is the LDA score—the greater the LDA score, the greater the influence of species abundance on the differential effect—and different colors represent different groups.

Principal coordinate analysis (PCoA) showed significant geographical differences between QH and DZ, and the microbial communities of the same or adjacent regions (stomach and small intestine, cecum, and rectum) were more similar than those in other regions (Figure 3A).

LEfSe was used to further determine whether the four sites of QH were differentially enriched for specific bacterial taxa compared to DZ. A cladogram representing the taxonomic hierarchy of the microbiota from phylum to species indicated significant differences in phylogenetic distribution among the eight groups of microbial communities (Figure 3B).

As indicated by the LDA plot in Figure 3C, when the LDA threshold was set to 4, the key discriminators were *Cyanobacteria* and *Chloroplast* in QH-Stomach; *Proteobacteria* and *Gammaproteobacteria* in QH-Small intestine; *Clostridia* and *Lachnospirales* in QH-Cecum; *Rikenellaceae* and *Lachnospiraceae*_AC2044_group in QH-Rectum; *Lactobacillales* and *Bacilli* in DZ-Small intestine; *Prevotellaceae* and *Spirochaetota* in DZ-Cecum; and *Bacteroidales* and *Bacterodia* in DZ-Rectum. These results indicated a significant difference in the microbiota composition of QH and DZ.

### Metagenomic analysis of functional pathways

After filtering, 729,568,690 reads and 110,083,165,328 bp were obtained. After assembly, 8,557,798 contigs were received, with an average length of 674,840,711 bp and N50 of 8,928 bp. Finally, 11,963,437 ORFs were generated. The pathways were analyzed using the eggNOG, Kyoto Encyclopedia of Genes and Genomes (KEGG), and CAZyme databases.

**Figure 4:**
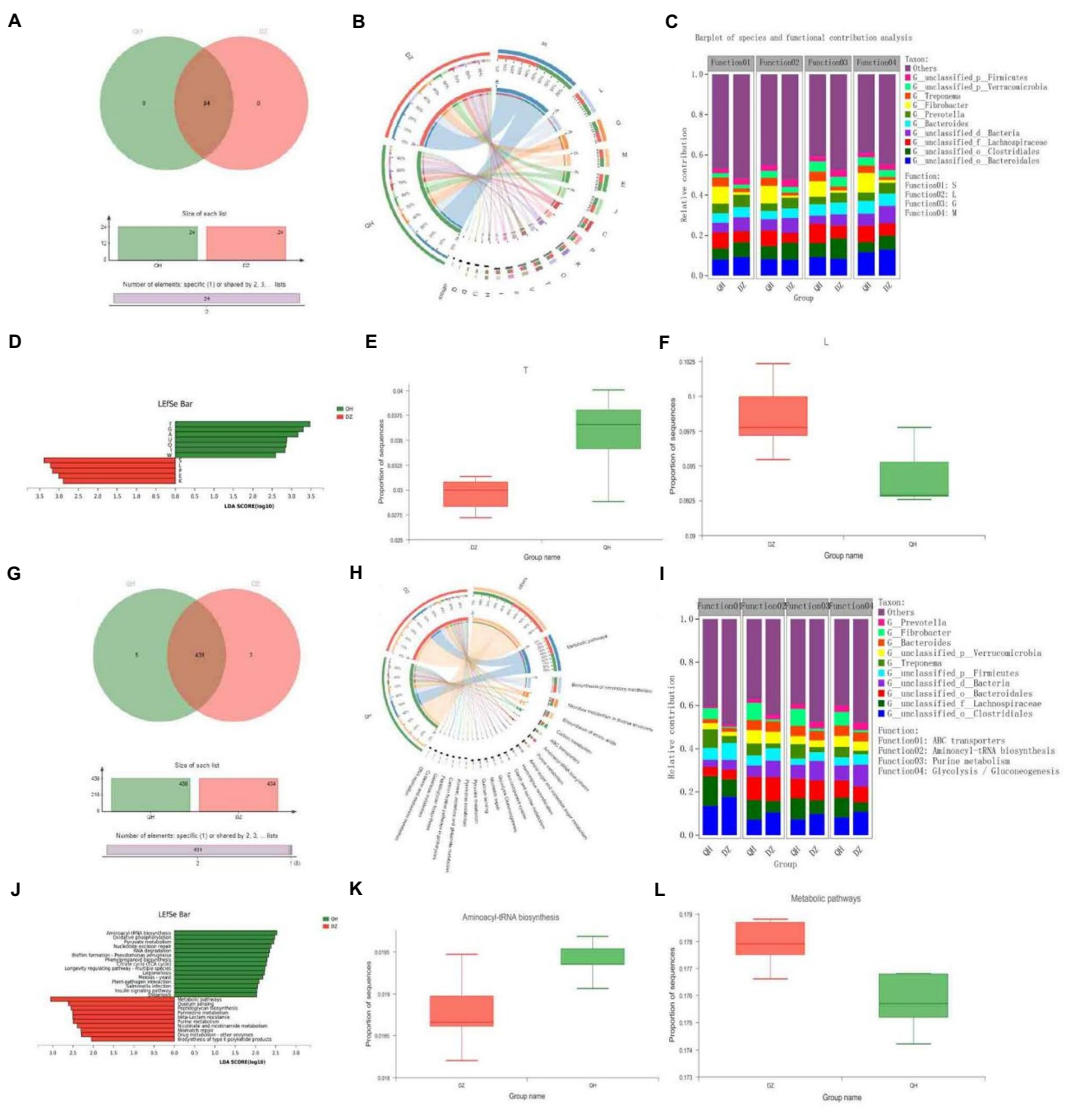
Functional composition and differences. Venn Figures in COG **(A)** and KEGG **(G)**. Overlapping parts indicate functions common to multiple sample groups; non-overlapping parts indicate functions unique to the sample group; and numbers indicate the number of corresponding functions. Circos Figures in COG **(B)** and KEGG **(H)**. The left semicircle (smaller circle) represents the functional abundance composition of the sample, and the right semicircle (larger circle) represents the distribution ratio of functions in different samples under this clustering-level condition. Species and functional contributions were shown in COG **(C)** and KEGG **(I)**; the abscissas were the corresponding sample groups, and the ordinates were the relative contributions. LDA discriminant histogram in COG **(D)** and KEGG **(J)**: the abscissa is the LDA score—the greater the LDA score, the greater the influence of species abundance on the differential effect—and different colors represent different groups. Difference test box in COG **(E,F)** and KEGG **(K,L)**: The abscissa represents the grouping category name, and the ordinate represents the percentage of species abundance in a sample grouping.

In the eggNOG function analysis, there were 24 COG functions were present in the QH and DZ groups (Figure 4A). In addition to function unknown [S], the most abundant functions were replication, recombination, and repair [L] (9.56%) and carbohydrate transport and metabolism [G] (8.04%) (Figure 4B). *Bacteroidales* were the major contributors to these functions in the QH group (Figure 4C). We identified 12 COGs (Figure 4D). Seven functional COG categories were identified in QH, including signal transduction mechanisms [T] (*p* = 0.0204) and carbohydrate transport and metabolism (Figure 4E). Five functional COG categories were in the DZ, except for function unknown [S], including replication, recombination, and repair [L] (*p* = 0.0249) (Figure 4F).

KEGG function analysis revealed 431 functions present in the QH and DZ groups (Figure 4G). The top two pathways with the highest abundance were metabolic pathways (17.68%) and biosynthesis of secondary metabolites (8.08%) (Figure 4H). In the KEGG species and function contribution picture, *Lachnospiraceae* had the highest contribution rate in QH, which was higher than that in DZ, whereas *Clostridiales* had the most abundant function in DZ, which was more than that in QH. Furthermore, *Clostridiales* and *Lachnospiraceae* contributed the most to the functional ATP-binding cassette (ABC) transporters (Figure 4I). In Figure 4J, 15 representative pathways were significantly enriched in QH, including aminoacyl-tRNA biosynthesis (*p* = 0.0430) (Figure 4K) and oxidative phosphorylation. Ten pathways were significantly enriched in DZ, including metabolic pathways (*p* = 0.0370) (Figure 4I), quorum sensing, and peptidoglycan biosynthesis.

**Figure 5:**
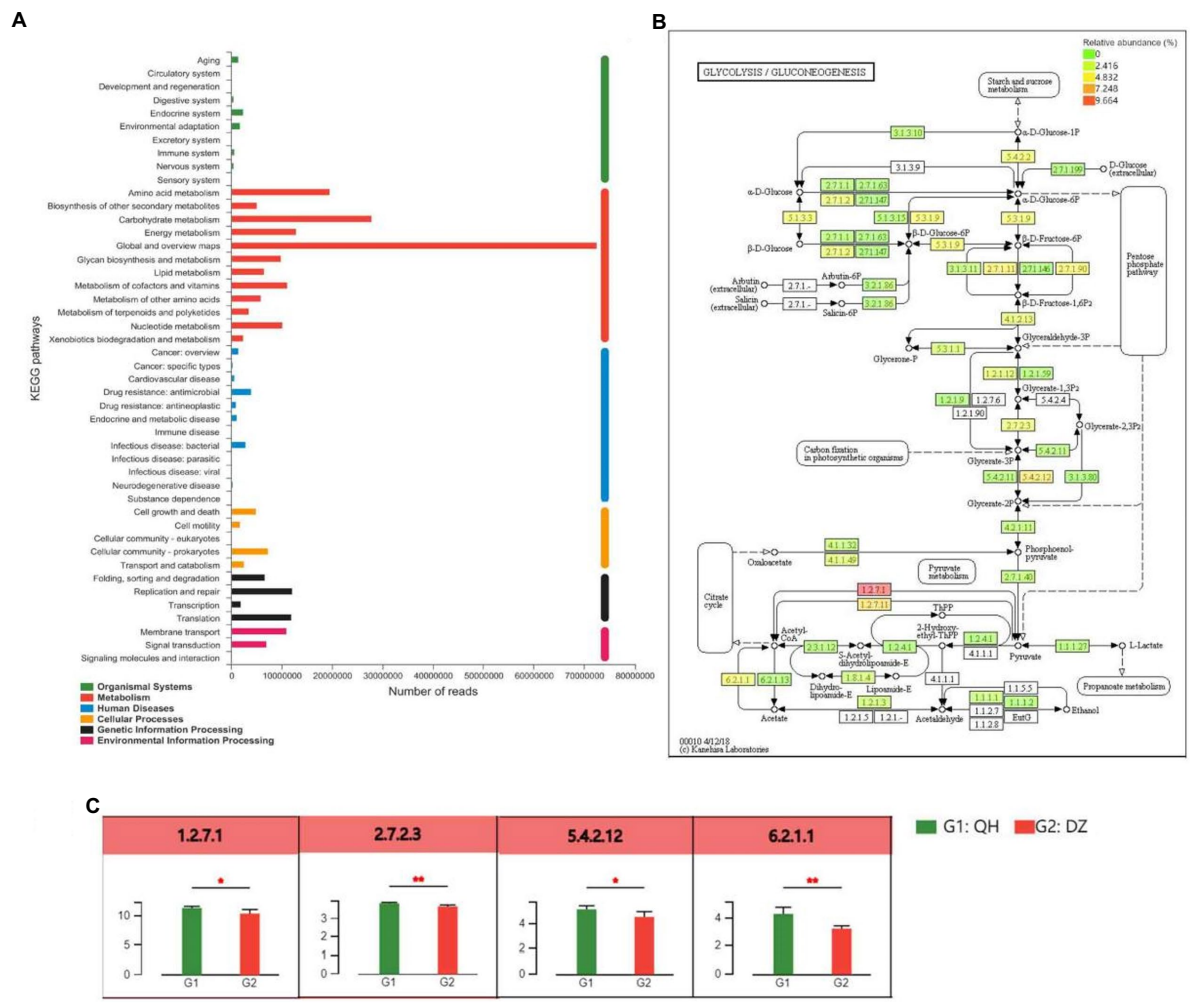
Differences between metabolite groups. **(A)** Pathway classification statistics histogram. The ordinate was the function name of KEGG Pathway Level 2, and the abscissa was its corresponding abundance value. The histogram was colored according to the KEGG Pathway Level 1 to which KEGG Pathway Level 2 belongs. **(B)** Metabolic pathway difference test chart between groups. Each square in the box with a fill color represents one or a group of samples. The intensity of the color represents the abundance change in the enzyme in different samples or groups. **(C)** Histogram of differences in metabolic pathways between groups. In the legend, G1 and G2 correspond to different groups.

Based on the KEGG annotation results, the differential detection and visual analysis of differentially abundant enzymes were performed for a certain pathway. Metabolism had the highest functional enrichment at level 2, and the top 3 functional abundances were global and overview maps, carbohydrate metabolism, and amino acid metabolism (Figure 5A). The most abundant carbohydrate pathway was glycolysis and gluconeogenesis, and differences in their metabolic pathways were shown in Figures 5B,C. Pyruvate ferredoxin oxidoreductase (EC:1.2.7.1, comprising four subunits, porA, porB, porG, and porD), 2,3-bisphosphoglycerate-independent phosphoglycerate mutase (EC:5.4.2.12, comprising three subunits, gpml, gpmB, and apgM) were higher in QH than in DZ (*p* < 0.05), phosphoglycerate kinase (PGK) (EC:2.7.2.3), and acetyl-CoA synthetase (EC:6.2.1.1, comprising 2 subunits of ACSS and AAE7) was higher than that of DZ in QH (*p* < 0.01).

## Discussion

The elevation of the Qinghai was higher than that of Dezhou, and the temperature and oxygen concentration decreased with an increase in elevation. The species composition of the gut flora was susceptible to environmental temperature, and decreased environmental temperature leads to a decrease in the alpha diversity of the flora (Chevalier et al., 2015; Moreno-Navarrete and Fernandez-Real, 2019). The alpha diversity of the gut flora of mice living at 12°C for a long time was significantly lower than that of mice living at 29°C (Zietak et al., 2016). However, other studies had found that the structure of the gut flora of mice living in a warm environment (22°C) was similar to that of mice living in a cold environment (4°C) (Li et al., 2019a,b). In addition, the community structure of the host gut flora remains stable in long-term hypoxic environments (Lucking et al., 2018; Khanna et al., 2021). Liu et al. (2020) analyzed the gut of Tibetan wild ass and African wild ass in the Qinghai region by using 16S rRNA genes sequencing; the results showed no significant difference in alpha diversity between the two groups, but the difference in beta diversity was significant. Tibetan wild ass had a relatively more complex bacterial network and a stronger dry matter digestion ability than the African wild ass. In this study, the diversity and richness of the flora of the Qinghai donkey were higher than those of the Dezhou donkey (Figure 1), indicating that the Qinghai donkey may have unique gut flora that can adapt to the hypoxic environment of the Tibetan Plateau.

At the phylum level (Figure 2C), the dominant flora of the Qinghai donkey were *Firmicutes*, *Proteobacteria*, and *Cyanobacteria* in the stomach; *Firmicutes* and *Proteobacteria* in the small intestine; and *Firmicutes* and *Bacteroidetes* in the cecum and rectum. We concluded that *Firmicutes* and *Proteobacteria* were the dominant phyla in the foregut of QH, and *Firmicutes* and *Bacteroidetes* were the dominant phyla in the hindgut of QH. The dominant flora of the Dezhou donkey were *Firmicutes* and *Bacteroidetes* in the stomach, *Firmicutes* in the small intestine, and *Firmicutes* and *Bacteroidetes* in the cecum and rectum. The abundance of *Firmicutes* in the Dezhou donkey foregut was higher than that in the hindgut, and that of *Bacteroidetes* in the hindgut was higher than that in the foregut. In equine animals, the foregut was mainly responsible for the digestion and absorption of food, with slight fermentation, and the hindgut was related to microbial fermentation, especially the large intestine (Argenzio et al., 1974, 1977; DiBaise et al., 2008). *Firmicutes* mainly uses carbohydrates in herbivores (Brulc et al., 2009), which can improve animal immunity and enhance gut function. *Bacteroides* have abundant genes encoding carbohydrate-active enzymes and can easily switch according to the availability of energy source types in the gut (Flint et al., 2012). *Bacteroides* were significantly enriched in the digestion-related microbiota of the large intestine and were not affected by the location of the gut in the mucosal-related microbiota. *Firmicutes* and *Bacteroides* were the two most dominant phyla among several other herbivores, such as goats (Li et al., 2019a,b), cattle (Mao et al., 2015), and horses (Al and Andrews, 2009). Previous research and this study suggest that the accumulation of *Firmicutes* and *Bacteroidetes* in the large intestine may help the host adapt to the complex internal environment.

At the genus level, *Lactobacillus* dominates the foregut, and *Streptococcus* dominates the hindgut. *Lactobacillus* can degrade fibrous carbohydrates (e.g., pentose, hexose, and starch), participate in the uncoupling of bile salts (Bao et al., 2012), and produce antimicrobial substances (e.g., bacteriocins and lactate) or compete with pathogens for mucosal adhesion sites and nutrients to inhibit the proliferation of pathogens (Umu et al., 2017). Lactate, the fermentation product of *Lactobacillus*, can acidify the gut mucosa. Research on horses found that *Lactobacillus* species dominated the stomach, whereas *Streptococcus* was significantly increased in the duodenum (Costa et al., 2015). In another study of DZ, the foregut microflora was dominated by *Lactobacillus*, and the hindgut microflora was dominated by *Streptococcus*, and *Lactobacillus* and *Streptococcus* were beneficial probiotics for equine animals (Liu et al., 2019).

In this study, the abundance of *Firmicutes*, *Fibrobacteres*, *Verrucomicrobia*, and *Spirochaetes* in the rectum was higher in QH than in DZ, and *Bacteroidetes* and *Clostridiales* were more abundant in the rectum of DZ than that of QH. Most bacteria in *Firmicutes* were butyrate-producing, and butyrate was one of the final metabolites of polysaccharides (Pryde et al., 2002). Polysaccharides entering the gut were crucial factors affecting the physiological state and composition of gut commensal bacteria. Butyric acid plays an important role in maintaining the integrity of rectal tissues and can prevent colon disease. Studies have shown that more than 35% of the enzymes required for animal digestion and metabolism were produced in the gut flora, and 25% of these enzymes were involved in carbohydrate metabolism (Gill et al., 2006). *Firmicutes* encode few carbohydrate-degrading enzymes but more ABC transporters (ATP-binding transporters) to transport carbohydrates (Mahowald et al., 2009). The most important carbohydrate transport system is the ATP-ABC-type transport system (Turroni et al., 2018). *Bacteroides*, the second most dominant group in the intestine, can degrade carbohydrates and provide the host with 10–15% of energy from food. Due to the efficient polysaccharide degradation system of *Bacteroidetes* and its ability to produce large amounts of short-chain fatty acids, *Bacteroidetes* has become the most studied strain in the transportation and utilization of polysaccharides in the intestinal flora, and approximately 20% of the genes in their genomes were used to complete the decomposition of sugar, also a possible reason why it had become a dominant strain (Singh, 2019). Jena et al. (2016) fed rats with a high-sugar diet for 60 days: the proportion of *Escherichia coli* and *Clostridium* in the intestinal tract increased, and the content of *Lactobacillus* decreased. They also investigated the composition of fecal microbiota and pro-inflammatory cytokines in serum by denaturing gradient gel electrophoresis. They found that the expression of genes such as TLR2, TLR4, and NF-kB increased in various tissues, and the inflammatory response in blood and tissues was enhanced, which significantly affected their metabolic status.

Besides, *Proteobacteria*, *Cyanobacteria*, *Actinobacteria*, and *chloroplasts* exist almost exclusively in the foregut of donkeys in the QH region. *Actinobacteria* affect various metabolic and physiological activities, including the production of extracellular enzymes, antibacterial activity, and formation of other secondary metabolites (Schrempf, 2001; Zhao et al., 2018). *Cyanobacteria* and *Chloroplasts* were involved in pathways related to photosynthesis (Crespo-Piazuelo et al., 2018). These flora became the potential biological markers to distinguish the two donkey breeds of QH and DZ.

The results of the present study suggest that QH had more pathways enriched in the glycolysis and gluconeogenesis pathways of carbohydrate metabolism than DZ did. The metabolic pathway results demonstrated that pyruvate ferredoxin oxidoreductase and acetyl-CoA synthetase participate in the citrate cycle, and PGK and 2,3-bisphosphoglycerate-independent phosphoglycerate mutase participate in the carbon fixation in photosynthetic organisms. In Methanosarcina species, ferredoxin was shown to be involved in methanogenesis from acetate (Ferry, 1993). H_2_ formation from pyruvate was stimulated by ferredoxin; thus, it may act as an electron carrier between pyruvate oxidoreductase and pyruvate hydrogenase (Hatchikian et al., 1982). All the tested archaea contained pyruvate ferredoxin oxidoreductase, which played a role in catabolism and anabolism. Thus, pyruvate ferredoxin oxidoreductase appears to represent the only mechanism for pyruvate acetyl-CoA conversion in the archaeal domain (Ikeda et al., 2006). Notably, 2,3-Diphosphoglycerate-independent phosphoglycerate mutase (IPGAM) catalyzes the reversible conversion of 3-phosphoglycerate (3-PGA) to 2-phosphoglycerate (2-PGA) during glycolysis key enzyme (Johnsen and Schonheit, 2007). There were two isoforms of PGK in the human genome, PGK1 and PGK2, with similar structures and functions, and more than 80% of the amino acid sequences were similar (McCarrey et al., 1992). PGK1 was the first key enzyme that generates ATP in the glycolytic pathway, and its participation in glycolysis was the main function of PGK1, which was important for the continuously production of cellular energy under hypoxic conditions (Sun et al., 2015). Acetyl-CoA was a central metabolite in carbon and energy metabolism. In mammalian cells, carbohydrates were converted into various biomolecules *via* many processes. Carbohydrates were first decomposed into acetyl-CoA, and then acetyl-CoA was used as a precursor for anabolism. This pathway was an important for the conversion of sugars into other biomolecules. The main synthesis pathways of mitochondrial acetyl-CoA comprise oxidative decarboxylation of the glycolysis product pyruvate, fatty acid β-oxidation, and branched-chain amino acid decomposition. Mitochondrial acetyl-CoA normally enters the tricarboxylic acid cycle for further metabolism (Boroughs and DeBerardinis, 2015). Although the available energy produced by gluconeogenesis and glycolysis was limited, this approach can supplement the much-needed energy for muscle hypoxia during heavy physical labor or long-term strenuous exercise, which was consistent with the environmental and service conditions of QH. The aforementioned analysis demonstrated that the main enriched metabolic pathways of QH donkeys were carbohydrate metabolism, and they were mainly enriched in glycolysis and gluconeogenesis pathways.

## Conclusion

In this study, we investigated the differences in gut microbes in QH from the Tibetan Plateau and DZ by using 16S rRNA gene high-throughput sequencing and metagenomic sequencing. The results showed that the flora diversity and richness of QH were higher than those of DZ, and the flora diversity and richness of the hindgut were higher than those of the foregut, with no sex difference. The major pathways associated with the Qinghai donkey were signal transduction mechanisms and carbohydrate transport and metabolism, and *Bacteroidetes* were the major contributor to these functions.

## Data availability statement

The datasets presented in this study can be found in online repositories. The names of the repository/repositories and accession number(s) can be found in the article/supplementary material.

## Ethics statement

The animal study was reviewed and approved by Animal Care and Use Committee of Qingdao Agricultural University.

## Author contributions

SL and YS conceived of and designed the experiments. SL, GZ, TX, SZ, JC, DA, JD and JS collected the samples. RG conducted the experiments. Samples were processed by RG, TX, JW and FZ. RG analyzed the data. RG wrote the original manuscript, and SL, YS, WS and QP contributed to writing the manuscript. All authors contributed to the article and approved the submitted version.

## Funding

This research was funded by the Shandong Province Science Major Project on Improved Agricultural Varieties, grant numbers 2013lz016 and 2017LZN022; Donkey Innovation Team of Shandong Modern Agricultural Industry Technology System, grant number SDAIT-27; Major Agricultural Application Technology Innovation Projects of Shandong Province, grant number SD2019 XM 008; Ministry of Agriculture and Rural Affairs “Construction of technical route for phenotypic identification of donkey skin and meat traits” (grant number 19211183); Experimental Technology Research Programme of Qingdao Agriculture University(20210021).

## Conflict of interest

The authors declare that the research was conducted in the absence of any commercial or financial relationships that could be construed as a potential conflict of interest.

## Publisher’s note

All claims expressed in this article are solely those of the authors and do not necessarily represent those of their affiliated organizations, or those of the publisher, the editors and the reviewers. Any product that may be evaluated in this article, or claim that may be made by its manufacturer, is not guaranteed or endorsed by the publisher.

## Footnotes

^1^ www.illumina.com

## References

[ref1] Al J. R. A. Andrews F. M. (2009). The bacterial community of the horse gastrointestinal tract and its relation to fermentative acidosis, laminitis, colic, and stomach ulcers. Vet. Clin. North Am. Equine Pract. 25, 199–215. doi: 10.1016/j.cveq.2009.04.005 19580934

[ref2] Argenzio R. A. Southworth M. Lowe J. E. Stevens C. E. (1977). Interrelationship of Na, HCO_3_, and volatile fatty acid transport by equine large intestine. Am. J. Phys. 233, E469–E478. 10.1152/ajpendo.1977.233.6.E469 596440

[ref3] Argenzio R. A. Southworth M. Stevens C. E. (1974). Sites of organic acid production and absorption in the equine gastrointestinal tract. Am. J. Phys. 226, 1043–1050. doi: 10.1152/ajplegacy.1974.226.5.1043, PMID: 4824856

[ref4] Bao Y. Wang Z. Zhang Y. Zhang J. Wang L.. (2012). Effect of lactobacillus plantarum P-8 on lipid metabolism in hyperlipidemic rat model. Eur. J. Lipid Sci. Technol. 114, 1230–1236. doi: 10.1002/ejlt.201100393

[ref5] Boroughs L. K. DeBerardinis R. J. (2015). Metabolic pathways promoting cancer cell survival and growth. Nat. Cell Biol. 17, 351–359. doi: 10.1038/ncb3124, PMID: 25774832 PMC4939711

[ref6] Brulc J. M. Antonopoulos D. A. Miller M. E. Wilson M. K. Yannarell A. C. Dinsdale E. A.. (2009). Gene-centric metagenomics of the fiber-adherent bovine rumen microbiome reveals forage specific glycoside hydrolases. Proc. Natl. Acad. Sci. U. S. A. 106, 1948–1953. doi: 10.1073/pnas.0806191105, PMID: 19181843 PMC2633212

[ref7] Chen S. Zhou Y. Chen Y. Gu J. (2018). Fastp: An ultra-fast all-in-one FASTQ preprocessor. Bioinformatics 34, i884–i890. doi: 10.1093/bioinformatics/bty560, PMID: 30423086 PMC6129281

[ref9] Chevalier C. Stojanovic O. Colin D. J. Suarez-Zamorano N. Tarallo V. Veyrat-Durebex C.. (2015). Gut microbiota orchestrates energy homeostasis during cold. Cell 163, 1360–1374. doi: 10.1016/j.cell.2015.11.004, PMID: 26638070

[ref10] Costa M. C. Silva G. Ramos R. V. Staempfli H. R. Arroyo L. G. Kim P.. (2015). Characterization and comparison of the bacterial microbiota in different gastrointestinal tract compartments in horses. Vet. J. 205, 74–80. doi: 10.1016/j.tvjl.2015.03.018, PMID: 25975855

[ref11] Crespo-Piazuelo D. Estelle J. Revilla M. Criado-Mesas L. Ramayo-Caldas Y. Ovilo C.. (2018). Characterization of bacterial microbiota compositions along the intestinal tract in pigs and their interactions and functions. Sci. Rep. 8:12727. doi: 10.1038/s41598-018-30932-6, PMID: 30143657 PMC6109158

[ref12] DiBaise J. K. Zhang H. Crowell M. D. Krajmalnik-Brown R. Decker G. A. Rittmann B. E. (2008). Gut microbiota and its possible relationship with obesity. Paper presented at the Mayo clinic proceedings, 83, 460–469, doi: 10.4065/83.4.460 18380992

[ref13] Edgar R. C. (2013). UPARSE: highly accurate OTU sequences from microbial amplicon reads. Nat. Methods 10, 996–998. doi: 10.1038/nmeth.2604, PMID: 23955772

[ref14] Egert M. de Graaf A. A. Smidt H. de Vos W. M. Venema K. (2006). Beyond diversity: functional microbiomics of the human colon. Trends Microbiol. 14, 86–91. doi: 10.1016/j.tim.2005.12.007, PMID: 16406528

[ref15] Ferry J. G. (1993). “Fermentation of acetate,” in Methanogenesis. Boston, MA: Springer, 304–334.

[ref16] Flint H. J. Scott K. P. Duncan S. H. Louis P. Forano E. (2012). Microbial degradation of complex carbohydrates in the gut. Gut Microbes 3, 289–306. doi: 10.4161/gmic.19897, PMID: 22572875 PMC3463488

[ref17] Fu L. Niu B. Zhu Z. Wu S. Li W. (2012). CD-HIT: accelerated for clustering the next-generation sequencing data. Bioinformatics 28, 3150–3152. doi: 10.1093/bioinformatics/bts565, PMID: 23060610 PMC3516142

[ref18] Gill S. R. Pop M. Deboy R. T. Eckburg P. B. Turnbaugh P. J. Samuel B. S.. (2006). Metagenomic analysis of the human distal gut microbiome. Science 312, 1355–1359. doi: 10.1126/science.1124234, PMID: 16741115 PMC3027896

[ref19] Hatchikian E. C. Bruschi M. Forget N. Scandellari M. (1982). Electron transport components from methanogenic bacteria: the ferredoxin from Methanosarcina barkeri (strain Fusaro). Biochem. Biophys. Res. Commun. 109, 1316–1323. doi: 10.1016/0006-291X(82)91921-0, PMID: 7168765

[ref20] He Y. Wu W. Zheng H. M. Li P. McDonald D. Sheng H. F.. (2018). Regional variation limits applications of healthy gut microbiome reference ranges and disease models. Nat. Med. 24, 1532–1535. doi: 10.1038/s41591-018-0164-x, PMID: 30150716

[ref21] Ikeda T. Ochiai T. Morita S. Nishiyama A. Yamada E. Arai H.. (2006). Anabolic five subunit-type pyruvate:ferredoxin oxidoreductase from Hydrogenobacter thermophilus TK-6. Biochem. Biophys. Res. Commun. 340, 76–82. doi: 10.1016/j.bbrc.2005.11.155, PMID: 16343420

[ref22] Jena P. K. Prajapati B. Mishra P. K. Seshadri S. (2016). Influence of gut microbiota on inflammation and pathogenesis of sugar rich diet induced diabetes. Immunome Res. 12, 109–119. doi: 10.4172/1745-7580.10000109

[ref23] Johnsen U. Schonheit P. (2007). Characterization of cofactor-dependent and cofactor-independent phosphoglycerate mutases from Archaea. Extremophiles 11, 647–657. doi: 10.1007/s00792-007-0094-x, PMID: 17576516

[ref24] Khanna K. Mishra K. P. Chanda S. Ganju L. Singh S. B. Kumar B. (2021). Effect of Synbiotics on amelioration of intestinal inflammation under hypobaric hypoxia. High Alt. Med. Biol. 22, 32–44. doi: 10.1089/ham.2020.0062, PMID: 33185493

[ref25] Lankau E. W. Hong P. Y. Mackie R. I. (2012). Ecological drift and local exposures drive enteric bacterial community differences within species of Galapagos iguanas. Mol. Ecol. 21, 1779–1788. doi: 10.1111/j.1365-294X.2012.05502.x, PMID: 22369350

[ref26] Li B. Li L. Li M. Lam S. M. Wang G. Wu Y.. (2019a). Microbiota depletion impairs thermogenesis of Brown adipose tissue and Browning of white adipose tissue. Cell Rep. 26, 2720–2737.e5. doi: 10.1016/j.celrep.2019.02.015, PMID: 30840893

[ref27] Li B. Zhang K. Li C. Wang X. Chen Y. Yang Y. (2019b). Characterization and comparison of microbiota in the gastrointestinal tracts of the goat (Capra hircus) during preweaning development. Front. Microbiol. 10. doi: 10.3389/fmicb.2019.02125, PMID: , 31572331 PMC6753876

[ref28] Li D. Liu C. M. Luo R. Sadakane K. Lam T. W. (2015). MEGAHIT: An ultra-fast single-node solution for large and complex metagenomics assembly via succinct de Bruijn graph. Bioinformatics 31, 1674–1676. doi: 10.1093/bioinformatics/btv033, PMID: 25609793

[ref29] Li R. Li Y. Kristiansen K. Wang J. (2008). SOAP: short oligonucleotide alignment program. Bioinformatics 24, 713–714. doi: 10.1093/bioinformatics/btn025, PMID: 18227114

[ref30] Liu G. Bou G. Su S. Xing J. Qu H. Zhang X.. (2019). Microbial diversity within the digestive tract contents of Dezhou donkeys. PLoS One 14:e0226186. doi: 10.1371/journal.pone.0226186, PMID: 31834903 PMC6910686

[ref31] Liu H. Zhao X. Han X. Xu S. Zhao L. Hu L.. (2020). Comparative study of gut microbiota in Tibetan wild asses (Equus kiang) and domestic donkeys (Equus asinus) on the Qinghai-Tibet plateau. PeerJ. 8:e9032. doi: 10.7717/peerj.9032, PMID: 32547852 PMC7276150

[ref32] Lorenc A. Linnenbrink M. Montero I. Schilhabel M. B. Tautz D. (2014). Genetic differentiation of hypothalamus parentally biased transcripts in populations of the house mouse implicate the Prader–Willi syndrome imprinted region as a possible source of behavioral divergence. Mol. Biol. 31, 3240–3249. doi: 10.1093/molbev/msu257, PMID: 25172960 PMC4245819

[ref33] Lucking E. F. O'Connor K. M. Strain C. R. Fouhy F. Bastiaanssen T. F. S. Burns D. P.. (2018). Chronic intermittent hypoxia disrupts cardiorespiratory homeostasis and gut microbiota composition in adult male Guinea-pigs. EBioMedicine 38, 191–205. doi: 10.1016/j.ebiom.2018.11.010, PMID: 30446434 PMC6306383

[ref34] Magoc T. Salzberg S. L. (2011). FLASH: fast length adjustment of short reads to improve genome assemblies. Bioinformatics 27, 2957–2963. doi: 10.1093/bioinformatics/btr507, PMID: 21903629 PMC3198573

[ref35] Mahowald M. A. Rey F. E. Seedorf H. Turnbaugh P. J. Fulton R. S. Wollam A.. (2009). Characterizing a model human gut microbiota composed of members of its two dominant bacterial phyla. Proc. Natl. Acad. Sci. 106, 5859–5864. doi: 10.1073/pnas.0901529106, PMID: 19321416 PMC2660063

[ref36] Mao S. Zhang M. Liu J. Zhu W. (2015). Characterising the bacterial microbiota across the gastrointestinal tracts of dairy cattle: membership and potential function. Sci. Rep. 5:16116. doi: 10.1038/srep16116, PMID: 26527325 PMC4630781

[ref38] McCarrey J. R. Berg W. M. Paragioudakis S. J. Zhang P. L. Dilworth D. D. Arnold B. L.. (1992). Differential transcription of Pgk genes during spermatogenesis in the mouse. Dev. Biol. 154, 160–168. doi: 10.1016/0012-1606(92)90056-M, PMID: 1426623

[ref39] Moeller A. H. Suzuki T. A. Lin D. Lacey E. A. Wasser S. K. Nachman M. W. (2017). Dispersal limitation promotes the diversification of the mammalian gut microbiota. Proc. Natl. Acad. Sci. U.S.A. 114, 13768–13773. doi: 10.1073/pnas.1700122114, PMID: 29229828 PMC5748161

[ref40] Monaghan T. M. Sloan T. J. Stockdale S. R. Blanchard A. M. Emes R. D. Wilcox M.. (2020). Metagenomics reveals impact of geography and acute diarrheal disease on the central Indian human gut microbiome. Gut Microbes 12:1752605. doi: 10.1080/19490976.2020.1752605, PMID: 32459982 PMC7781581

[ref41] Moreno-Navarrete J. M. Fernandez-Real J. M. (2019). The gut microbiota modulates both browning of white adipose tissue and the activity of brown adipose tissue. Rev. Endocr. Metab. Disord. 20, 387–397. doi: 10.1007/s11154-019-09523-x, PMID: 31776853

[ref42] Noguchi H. Park J. Takagi T. (2006). MetaGene: prokaryotic gene finding from environmental genome shotgun sequences. Nucleic Acids Res. 34, 5623–5630. doi: 10.1093/nar/gkl723, PMID: 17028096 PMC1636498

[ref43] Phillips C. D. Phelan G. Dowd S. E. McDonough M. M. Ferguson A. W. Delton H. J.. (2012). Microbiome analysis among bats describes influences of host phylogeny, life history, physiology and geography. Mol. Ecol. 21, 2617–2627. doi: 10.1111/j.1365-294X.2012.05568.x, PMID: 22519571

[ref44] Pryde S. E. Duncan S. H. Hold G. L. Stewart C. S. Flint H. J. (2002). The microbiology of butyrate formation in the human colon. FEMS Microbiol. Lett. 217, 133–139. doi: 10.1111/j.1574-6968.2002.tb11467.x 12480096

[ref45] Rothschild D. Weissbrod O. Barkan E. Kurilshikov A. Korem T. Zeevi D.. (2018). Environment dominates over host genetics in shaping human gut microbiota. Nature 555, 210–215. doi: 10.1038/nature25973, PMID: 29489753

[ref46] Schrempf H. (2001). Recognition and degradation of chitin by streptomycetes. Antonie Van Leeuwenhoek 79, 285–289. doi: 10.1023/A:1012058205158 11816971

[ref47] Singh R. P. (2019). Glycan utilisation system in Bacteroides and Bifidobacteria and their roles in gut stability and health. Appl. Microbiol. Biotechnol. 103, 7287–7315. doi: 10.1007/s00253-019-10012-z, PMID: 31332487

[ref48] Stackebrandt E. Goebel B. M. (1994). Taxonomic note: a place for DNA-DNA reassociation and 16S rRNA sequence analysis in the present species definition in bacteriology. Int. J. Syst. Evol. Microbiol. 44, 846–849. doi: 10.1099/00207713-44-4-846

[ref49] Sudakaran S. Salem H. Kost C. Kaltenpoth M. (2012). Geographical and ecological stability of the symbiotic mid-gut microbiota in European firebugs, Pyrrhocoris apterus (Hemiptera, Pyrrhocoridae). Mol. Ecol. 21, 6134–6151. doi: 10.1111/mec.12027, PMID: 23017151

[ref50] Sullam K. E. Essinger S. D. Lozupone C. A. O'Connor M. P. Rosen G. L. Knight R.. (2012). Environmental and ecological factors that shape the gut bacterial communities of fish: a meta-analysis. Mol. Ecol. 21, 3363–3378. doi: 10.1111/j.1365-294X.2012.05552.x, PMID: 22486918 PMC3882143

[ref51] Sun S. Liang X. Zhang X. Liu T. Shi Q. Song Y.. (2015). Phosphoglycerate kinase-1 is a predictor of poor survival and a novel prognostic biomarker of chemoresistance to paclitaxel treatment in breast cancer. Br. J. Cancer 112, 1332–1339. doi: 10.1038/bjc.2015.114, PMID: 25867275 PMC4402453

[ref52] Turroni F. Milani C. Duranti S. Mahony J. van Sinderen D. Ventura M. (2018). Glycan utilization and cross-feeding activities by bifidobacteria. Trends Microbiol. 26, 339–350. doi: 10.1016/j.tim.2017.10.001, PMID: 29089173

[ref53] Umu O. C. O. Rudi K. Diep D. B. (2017). Modulation of the gut microbiota by prebiotic fibres and bacteriocins. Microb. Ecol. Health Dis. 28:1348886. doi: 10.1080/16512235.2017.1348886, PMID: 28959178 PMC5614387

[ref54] Wang Q. Garrity G. M. Tiedje J. M. Cole J. R. (2007). Naive Bayesian classifier for rapid assignment of rRNA sequences into the new bacterial taxonomy. Appl. Environ. Microbiol. 73, 5261–5267. doi: 10.1128/AEM.00062-07, PMID: 17586664 PMC1950982

[ref55] Zhao X. Zhang X. Chen Z. Wang Z. Lu Y. Cheng D. (2018). The divergence in bacterial components associated with Bactrocera dorsalis across developmental stages. Front. Microbiol. 9:114. doi: 10.3389/fmicb.2018.00114, PMID: 29449838 PMC5799270

[ref56] Zhou X. Jiang X. Yang C. Ma B. Lei C. Xu C.. (2016). Cecal microbiota of Tibetan chickens from five geographic regions were determined by 16S rRNA sequencing. Microbiology 5, 753–762. doi: 10.1002/mbo3.367, PMID: 27139888 PMC5061713

[ref57] Zietak M. Kovatcheva-Datchary P. Markiewicz L. H. Stahlman M. Kozak L. P. Backhed F. (2016). Altered microbiota contributes to reduced diet-induced obesity upon cold exposure. Cell Metab. 23, 1216–1223. doi: 10.1016/j.cmet.2016.05.001, PMID: 27304513 PMC4911343

